# Supplementary material for: A Proposed Saffron Soilless Cultivation System for a Quality Spice as Certified by Genetic Traceability
Source: Plants (Basel). 2024 Dec 27;14(1):51. doi: 10.3390/plants14010051 (PMC11723413; doi:10.3390/plants14010051)
Supplement: Supplementary file 1 [file plants-14-00051-s001.zip › Figure S1.pdf]

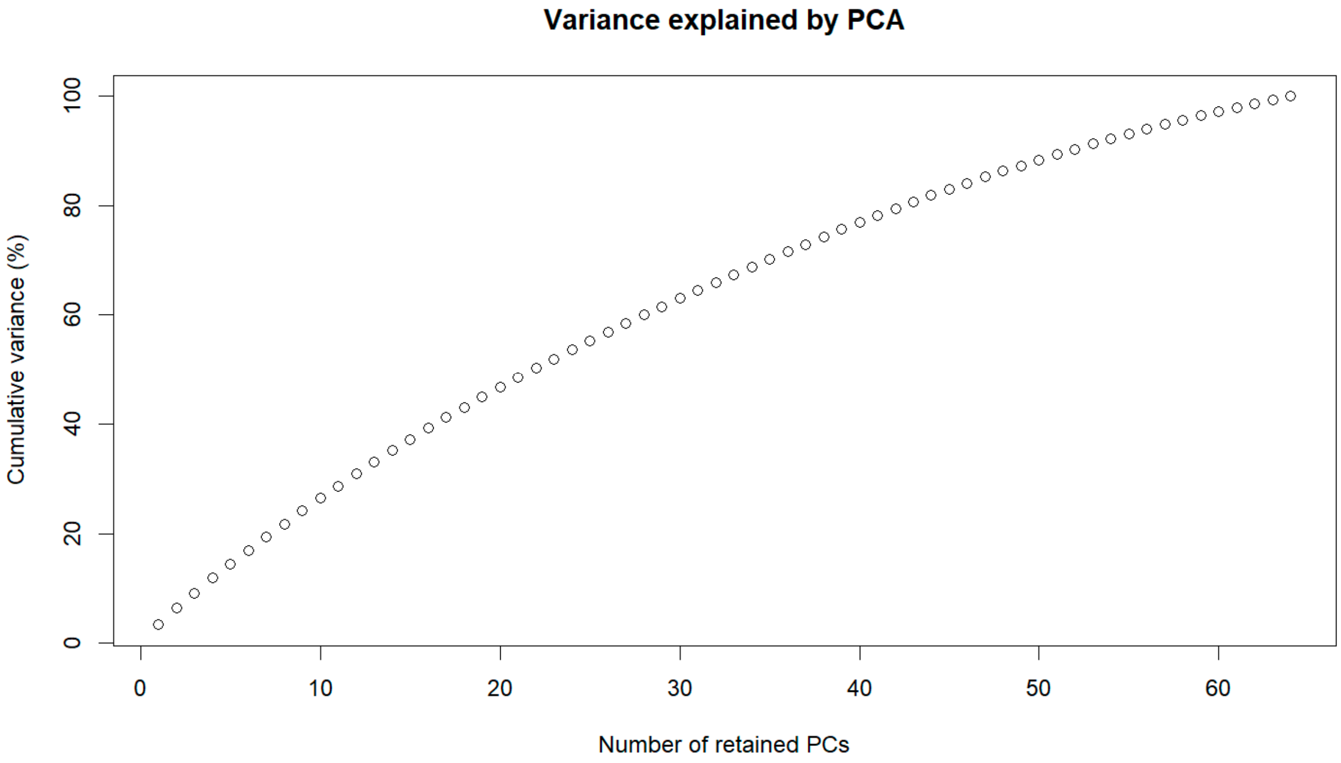

Figure S1 –Variance retained by the Principal Components (PCs) in the Principal Component Analysis (PCA) step of the Discriminant Analysis of Principal Components (DAPC).
