# Supplementary material for: A Proposed Saffron Soilless Cultivation System for a Quality Spice as Certified by Genetic Traceability
Source: Plants (Basel). 2024 Dec 27;14(1):51. doi: 10.3390/plants14010051 (PMC11723413; doi:10.3390/plants14010051)
Supplement: Supplementary file 1 [file plants-14-00051-s001.zip › Figure S3.pdf]

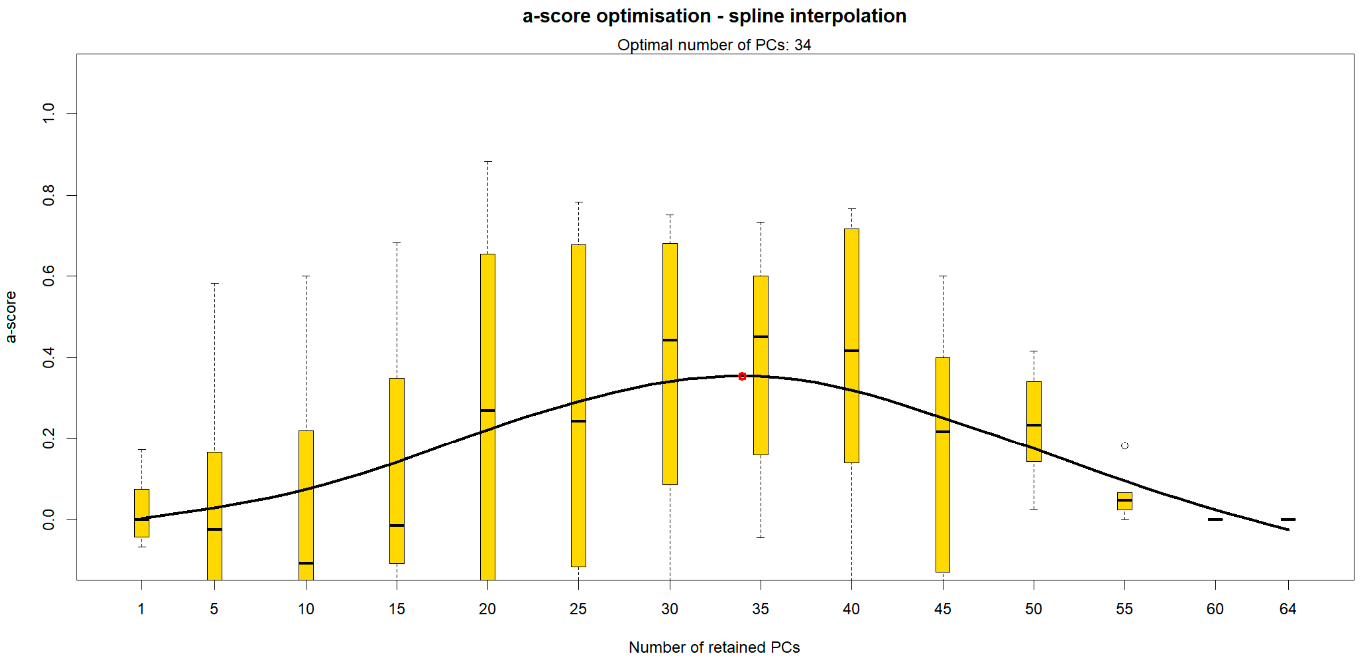

Figure S3 – Optimal a-score from the Discriminant Analysis of Principal Components (DAPC). The red dot indicates the best number of Principal Components (PCs) to retain in the analysis.
