# Supplementary material for: A Proposed Saffron Soilless Cultivation System for a Quality Spice as Certified by Genetic Traceability
Source: Plants (Basel). 2024 Dec 27;14(1):51. doi: 10.3390/plants14010051 (PMC11723413; doi:10.3390/plants14010051)
Supplement: Supplementary file 1 [file plants-14-00051-s001.zip › Table S1.pdf]

Table S1 – Plant material included in population genetics study.

	Sample Code	Accession	Site of Origin	Country	Collection
**Z_1**	MOR_3	RO.LO.	Foligno (PERUGIA)	ITALY	DSA3
**Z_2**	MOR_4	RO.LO.	Foligno (PERUGIA)	ITALY	DSA3
**Z_3**	MOR_5	RO.LO.	Foligno (PERUGIA)	ITALY	DSA3
**Z_4**	VEN_2	Venturi	Gualdo Tadino (PERUGIA)	ITALY	DSA3
**Z_5**	VEN_4	Venturi	Gualdo Tadino (PERUGIA)	ITALY	DSA3
**Z_6**	VEN_5	Venturi	Gualdo Tadino (PERUGIA)	ITALY	DSA3
**Z_7**	FIN_1	Porta Sole	Perugia	ITALY	DSA3
**Z_8**	FIN_2	Porta Sole	Perugia	ITALY	DSA3
**Z_9**	FIN_5	Porta Sole	Perugia	ITALY	DSA3
**Z_10**	MAZ_1	Mazzuoli	Città della Pieve (PERUGIA)	ITALY	DSA3
**Z_11**	MAZ_2	Mazzuoli	Città della Pieve (PERUGIA)	ITALY	DSA3
**Z_12**	MAZ_4	Mazzuoli	Città della Pieve (PERUGIA)	ITALY	DSA3
**Z_13**	GNP_2	Zafferano&Dintorni	Sant'Anatolia di Narco (PERUGIA)	ITALY	DSA3
**Z_14**	GNP_3	Zafferano&Dintorni	Sant'Anatolia di Narco (PERUGIA)	ITALY	DSA3
**Z_15**	GNP_4	Zafferano&Dintorni	Sant'Anatolia di Narco (PERUGIA)	ITALY	DSA3
**Z_16**	VNB_2	Vinerbi	Città della Pieve (PERUGIA)	ITALY	DSA3
**Z_17**	VNB_3	Vinerbi	Città della Pieve (PERUGIA)	ITALY	DSA3
**Z_18**	VNB_4	Vinerbi	Città della Pieve (PERUGIA)	ITALY	DSA3
**Z_19**	LIP_1	Alfonsi	Nocera Umbra (PERUGIA)	ITALY	DSA3
**Z_20**	LIP_2	Alfonsi	Nocera Umbra (PERUGIA)	ITALY	DSA3
**Z_21**	LIP_4	Alfonsi	Nocera Umbra (PERUGIA)	ITALY	DSA3
**Z_22**	BRS_1	Barisciano	Barisciano (L'AQUILA)	ITALY	DSA3
**Z_23**	BRS_3	Barisciano	Barisciano (L'AQUILA)	ITALY	DSA3
**Z_24**	BRS_4	Barisciano	Barisciano (L'AQUILA)	ITALY	DSA3
**Z_25**	BSC_1	Busconi		ITALY	DSA3
**Z_26**	BSC_2	Busconi		ITALY	DSA3
**Z_27**	BSC-3	Busconi		ITALY	DSA3
**Z_28**	TURC_1	BCU002472	Davutobasi (Safranbolu)	TURKEY	WSCC
**Z_29**	TURC_3	BCU002472	Davutobasi (Safranbolu)	TURKEY	WSCC
**Z_30**	TURC_4	BCU002472	Davutobasi (Safranbolu)	TURKEY	WSCC
**Z_31**	IRANa_1	BCU001695	Ferdos (Khorasan)	IRAN	WSCC
**Z_32**	IRANa_2	BCU001695	Ferdos (Khorasan)	IRAN	WSCC
**Z_33**	IRANa_4	BCU001695	Ferdos (Khorasan)	IRAN	WSCC
**Z_34**	IND_2	WSCC	Amelia	INDIA	WSCC
**Z_35**	IND_3	WSCC	Amelia	INDIA	WSCC
**Z_36**	IND_4	WSCC	Amelia	INDIA	WSCC
**Z_37**	FRNa_2	BCU002479	Carsaj (Pyrenees)	FRANCE	WSCC
**Z_38**	FRNa_3	BCU002479	Carsaj (Pyrenees)	FRANCE	WSCC
**Z:39**	FRNa_4	BCU002479	Carsaj (Pyrenees)	FRANCE	WSCC
**Z_40**	FRNb_1	BCU002864		FRANCE	WSCC
**Z_41**	FRNb_2	BCU002864		FRANCE	WSCC
**Z_42**	FRNb_4	BCU002864		FRANCE	WSCC
**Z_43**	SPGa_1	BCU001672	Monreal del Campo (Aragon)	SPAIN	WSCC
**Z_44**	SPGa_4	BCU001672	Monreal del Campo (Aragon)	SPAIN	WSCC
**Z_45**	SPGa_3	BCU001672	Monreal del Campo (Aragon)	SPAIN	WSCC
**Z_46**	SPGb_1	BCU001584	Minaya (Castillia la Mancha)	SPAIN	WSCC
**Z_47**	SPGb_3	BCU001584	Minaya (Castillia la Mancha)	SPAIN	WSCC
**Z_48**	SPGb_14	BCU001584	Minaya (Castillia la Mancha)	SPAIN	WSCC
**Z_49**	ARG_1	BCU001751	Valle dell'Uco (Cuyo)	ARGENTINA	WSCC
**Z_50**	ARG_2	BCU001751	Valle dell'Uco (Cuyo)	ARGENTINA	WSCC

	Sample Code	Accession	Site of Origin	Country	Collection
**Z_51**	ARG_3	BCU001751	Valle dell'Uco (Cuyo)	ARGENTINA	WSCC
**Z_52**	GRCa_1	BCU002911	Ano komi (Macedonia)	GREECE	WSCC
**Z_53**	GRCa_3	BCU002911	Ano komi (Macedonia)	GREECE	WSCC
**Z_54**	GRCa_4	BCU002911	Ano komi (Macedonia)	GREECE	WSCC
**Z_55**	GRCb_1	BCU002930	Maguola (Macedonia)	GREECE	WSCC
**Z_56**	GRCb_1	BCU002930	Maguola (Macedonia)	GREECE	WSCC
**Z_57**	GRCb_1	BCU002930	Maguola (Macedonia)	GREECE	WSCC
**Z_58**	GG_1	GG	Gorgain	IRAN	WSCC
**Z_59**	GG_3	GG	Gorgain	IRAN	WSCC
**Z_60**	GG_5	GG	Gorgain	IRAN	WSCC
**Z_61**	4_10_3	4_10	Ghasabeh (Khorasan)	IRAN	WSCC
**Z_62**	4_10_4	4_10	Ghasabeh (Khorasan)	IRAN	WSCC
**Z_63**	8_15_1	8_15	Gonabad (Khorasan)	IRAN	WSCC
**Z_64**	8_15_3	8_15	Gonabad (Khorasan)	IRAN	WSCC
**Z_66**	4_3_1	4_3	Ghasabeh	IRAN	WSCC
