# Supplementary material for: A Proposed Saffron Soilless Cultivation System for a Quality Spice as Certified by Genetic Traceability
Source: Plants (Basel). 2024 Dec 27;14(1):51. doi: 10.3390/plants14010051 (PMC11723413; doi:10.3390/plants14010051)
Supplement: Supplementary file 1 [file plants-14-00051-s001.zip › Table S2.pdf]

Table S2 – Barcodes, index sequences, and filtered Illumina read counts for each analyzed sample.

Sample name	Barcode_seq	Illumina index	Filtered Reads
Z1	GCTACAA	Idx_2_CGATGT	9093725
Z2	AGCATCA		16069770
Z3	CCATCCA		14889281
Z4	ACTGTAA		3998848
Z5	AGAGATA		11393109
Z6	AGCCACG		12012468
Z7	AAGCACA		10341690
Z8	ATCAGCA		9815466
Z9	CACATAT		5523581
Z10	GCTACAA	Idx_4_TGACCA	5554811
Z11	AGCATCA		9123167
Z12	CCATCCA		11048488
Z13	ACTGTAA		1790796
Z14	AGAGATA		3358189
Z15	AGCCACG		6642608
Z16	AAGCACA		5173302
Z17	ATCAGCA		4128114
Z18	CACATAT		4595495
Z19	GCTACAA	Idx_5_ACAGTG	9845729
Z20	AGCATCA		11852332
Z21	CCATCCA		11066255
Z22	ACTGTAA		2830545
Z23	AGAGATA		8436474
Z24	AGCCACG		8953207
Z25	AAGCACA		11607020
Z26	ATCAGCA		7835456
Z27	CACATAT		4361255
Z28	GCTACAA	Idx_6_GCCAAT	7083505
Z29	AGCATCA		10309046
Z30	CCATCCA		10357409
Z31	ACTGTAA		2255406
Z32	AGAGATA		5771121
Z33	AGCCACG		7787105
Z34	AAGCACA		3319355
Z35	ATCAGCA		6083916
Z36	CACATAT		4705964
Z37	GCTACAA	Idx_7_CAGATC	7285229
Z38	AGCATCA		9896583
Z39	CCATCCA		11323651
Z40	ACTGTAA		4489817
Z41	AGAGATA		7058590
Z42	AGCCACG		7684177
Z43	AAGCACA		9601175
Z44	ATCAGCA		6228576
Z45	CACATAT		4275364

Sample name	Barcode_seq	Illumina index	Filtered Reads
Z46	GCTACAA	Idx_12_CTTGTA	10288291
Z47	AGCATCA		8499985
Z48	CCATCCA		13072104
Z49	ACTGTAA		2601650
Z50	AGAGATA		6106500
Z51	AGCCACG		7039358
Z52	AAGCACA		6328541
Z53	ATCAGCA		3302101
Z54	CACATAT		5286380
Z55	GCTACAA	Idx_14_AGTTCC	9622303
Z56	AGCATCA		11348904
Z57	CCATCCA		10383414
Z58	ACTGTAA		3083652
Z59	AGAGATA		5944753
Z60	AGCCACG		7770268
Z61	AAGCACA		6968889
Z62	ATCAGCA		6848033
Z63	CACATAT		4373186
Z64	GCTACAA	Idx_15_ATGTCA	3320830
Z66	CCATCCA		2741067
